# Aortopexy Complicated by ST Segment Elevations in a Four-Month-Old Infant

**DOI:** 10.7759/cureus.11436

**Published:** 2020-11-11

**Authors:** Rita Saynhalath, Rhae Battles, Sonia D Mehta, Gijo Alex

**Affiliations:** 1 Anesthesiology and Pain Management, University of Texas Southwestern Medical Center, Dallas, USA; 2 Outcomes Research Consortium, Cleveland, USA; 3 Anesthesiology, University of Florida, Gainesville, USA

**Keywords:** cardiac arrhythmias, electrophysiology, echocardiography, infant, tracheomalacia

## Abstract

A four-month-old female infant presented for a thoracoscopic aortopexy for severe tracheomalacia. The case proceeded uneventfully until a specimen bag was introduced into the chest to remove the thymus. The child developed significant ST segment elevations in all 12 leads on electrocardiogram. An emergent intraoperative echocardiogram was performed but did not reveal any findings to account for the diffuse ST segment elevations. The ST segment elevations remained elevated for 48 hours following the procedure with no apparent hemodynamic instability or structural damage to the heart. Troponin levels returned to normal a few days later, and the child was discharged home without sequelae. The incidence of ST segment elevations in children without congenital heart disease is rare but can potentially lead to significant morbidity and mortality. This case report seeks to highlight the importance of swift recognition of ST changes in the pediatric population, discuss possible causes, and describe appropriate workup.

## Introduction

The pediatric population is generally at low risk for cardiac complications, especially children who do not have congenital heart disease. Myocardial ischemic changes develop when there is a sudden reduction or interruption of blood flow to the coronary vessels. If the coronary vessels are not rapidly re-channeled or re-vascularized, then a myocardial infarction (MI) develops. The electrocardiographic (EKG), echocardiographic, and enzymatic diagnostic criteria of MI have been well defined in adults, but in children there are some difficulties.

Gazit et al. describe three pediatric cases in which an EKG was used to diagnose a transmural acute MI, but further evaluation revealed coronary artery abnormalities in only one patient [1]. Geyer et al. reported on two patients who experienced ST segment changes intraoperatively, but in both cases, the EKG changes resolved by the end of surgery and the patients suffered no other complications [2].

This case report describes unanticipated ST segment elevations during a thoracoscopic aortopexy in an infant. We aim to discuss the appropriate workup for ST segment changes in the pediatric population and possible causes as well as management if the diagnosis of MI is confirmed. We obtained parental consent for our case report.

## Case presentation

We present the case of a four-month-old female infant who underwent a thoracoscopic aortopexy for severe tracheomalacia. The patient was born at 34 weeks with a two-vessel cord and a history of vertebral defects, anal atresia, cardiac defects, tracheoesophageal fistula, renal anomalies, and limb abnormalities (VACTERL) presenting with thoracic vertebral anomalies, a ventricular septal defect (VSD), a tracheoesophageal fistula with esophageal atresia status postrepair and gastrostomy tube placement, severe bronchopulmonary dysplasia, and retinopathy of prematurity. She had suffered a cardiopulmonary arrest due to tracheomalacia-induced hypoxia. Therefore, we decided to proceed with a bronchoscopy and thoracoscopic aortopexy.

**Figure 1:**
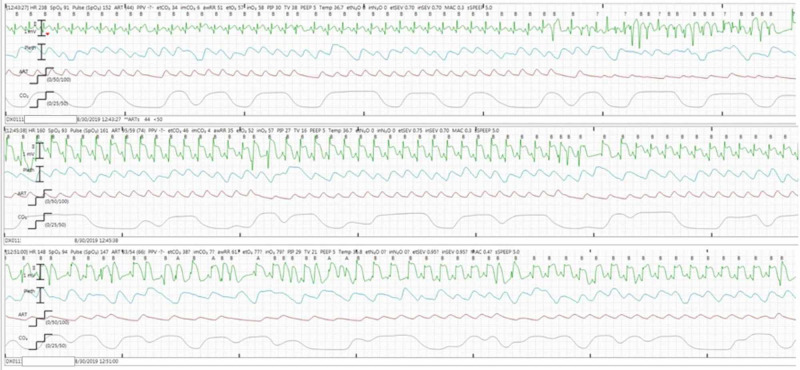
Intraoperative electrocardiography showing diffuse ST segment elevations.

A preoperative echocardiogram was significant for a small perimembranous VSD with left-to-right interventricular shunt; both ventricles were normal in size and function. The surgery proceeded uneventfully, including thymus dissection and aortopexy, until a large specimen bag was introduced into the chest to remove the thymus prior to chest closure. At that point, ST segment elevations were noted on the monitor and persisted despite removing the bag. The patient’s blood pressure, oxygen saturation, and end-tidal carbon dioxide concentration remained stable. The inhaled fraction of oxygen was increased to 100% while an intraoperative cardiology consultation was obtained. ST segment elevations were seen in leads I, II, and III. When additional EKG leads were placed on the patient’s chest to obtain a 12-lead EKG, diffuse ST segment elevations were seen throughout (Figure 1).

The patient remained hemodynamically stable despite the sustained ST segment elevations. Laboratory workup including an arterial blood gas and an electrolyte panel both returned within normal limits. The echocardiogram revealed normal coronary blood flow and normal to hyperdynamic systolic function of the left ventricle. The right ventricle was normal in size and systolic function. Electrophysiology was also consulted and diagnosed short episodes of non-sustained ventricular tachycardia, which had started shortly after thoracoscopic insufflation. A lidocaine infusion was initiated to prevent further arrhythmias. The surgical team closed the chest incisions, and the patient was transferred to the pediatric intensive care unit, intubated, and sedated. A postoperative echocardiogram revealed normal coronary artery structures, normal biventricular and size function, and a tiny VSD. The serum troponin level peaked immediately postoperatively at 24 ng/mL (normal serum level < 0.1 ng/mL) but continued to trend downward. No further episodes of arrhythmia occurred. Over the next few weeks, the patient remained hemodynamically stable with no additional cardiac complications. She was eventually extubated and discharged home with no apparent lasting cardiac sequelae.

## Discussion

The workup for EKG changes in the pediatric patient is challenging, given the presumed low frequency of cardiac events in this population. The actual incidence of MI is unknown and likely underestimated due to the lack of established diagnostic criteria [3]. Signs and symptoms of myocardial ischemia or infarction in children differ from those in adults. Furthermore, when these EKG changes occur under general anesthesia, typically only the physiologic parameters can be used to evaluate the etiology of ST changes.

Gorla et al. conducted a retrospective chart review to evaluate the association between abnormalities on EKG, such as ST-T wave changes, and perinatal course and congenital heart disease. He concluded that such findings are commonly observed in normal and stressed neonates and do not predict critical congenital heart disease. In comparison, similar changes found in older children and adults would be highly suspicious for myocardial ischemia and inflammation [4].

Gazit et al. also presented a pediatric case series to illustrate the challenges in diagnosing myocardial ischemia in children. First, tests that are commonly and accurately used in the adult population have an indeterminate sensitivity and specificity in pediatric patients. Second, there is a broad list of differential diagnoses when ischemia is suspected from EKG tracings. They include non-cardiac etiologies such as infections, neoplasms, inflammatory vascular diseases, genetic disorders, and trauma [1]. Finally, in the pediatric population, certain structural anomalies of the heart present as ST segment changes for the first time while under the stress of general anesthesia. Cardiac etiologies that should be ruled out include anomalous left coronary artery from the pulmonary artery and pulmonary arteriovenous fistulas [5].

Appropriate workup following ST segment changes under general anesthesia should include electrolyte and troponin laboratory studies, a full 12-lead EKG, and an echocardiogram to exclude anatomic abnormalities. A cardiology consultation is necessary to assist in the decision tree and discussion regarding coronary angiography. It is important to note that ST segment changes can be observed with only mild elevations of serum troponin levels without any evidence of coronary artery occlusion on angiogram [1]. Management of a confirmed MI includes hemodynamic stabilization; anticoagulation; and prevention of congestive heart failure with beta-blockers, angiotensin-converting enzyme inhibitors, diuretics, vasodilators, and calcium-channel blockers [3].

Towbin et al. sought to establish more specific EKG criteria to assist in the diagnosis of acute MI in children. This was done through a retrospective review of the EKGs of 37 patients younger than 21 years of age in which autopsy analysis demonstrated transmural MI. The five criteria were (1) new appearance of wide Q waves > 35 ms in duration, (2) increased amplitude or duration (>35 ms) of pre-existing Q waves, (3) new-onset Q waves in serial tracings, (4) Q-wave notching, and (5) ST segment elevation greater than or equal to 2 mm and prolonged QTc > 440 ms when associated with any other criterion. The study found that wide Q waves lasting greater than 35 ms was the most specific finding for the diagnosis of a transmural MI [6].

## Conclusions

Based on the diagnostic workup and the hospital course of our patient, the etiology for the prolonged ST segment elevations and transient elevation in serum troponin levels remains unclear. Interestingly, serial EKGs did not reveal Q wave abnormalities, and the ST changes resolved eventually. The list of differential diagnoses includes myocardial ischemia, irritation of the pericardium from the specimen bag, mechanical obstruction, and metabolic disturbance. The most likely explanation given the patient’s normal cardiac anatomy is transient disruption of the coronary blood flow when the large specimen bag was introduced into the small intrathoracic cavity.

## References

[1] Gazit AZ, Avari JN, Balzer DT, Rhee EK (2007). Electrocardiographic diagnosis of myocardial ischemia in children: is a diagnostic electrocardiogram always diagnostic?. Pediatrics.

[2] Geyer ED, Cartabuke RS, Schloss B, Tobias JD (2018). Intraoperative ST segment, T wave changes in two infants during general anesthesia. Anaesthesia Pain & Intensive Care.

[3] Papneja K, Chan AK, Mondal TK, Paes B (2017). Myocardial infarction in neonates: a review of an entity with significant morbidity and mortality. Pediatr Cardiol.

[4] Gorla SR, Hsu DT, Kulkarni A (2016). Lack of association of ST-T wave abnormalities to congenital heart disease in neonates. Congenit Heart Dis.

[5] Alfirevic A, Mossad E, Niezgoda J (2005). Unexpected ST segment changes in children - a case report. Paediatr Anaesth.

[6] Towbin JA, Bricker JT, Garson Jr A (1992). Electrocardiographic criteria for diagnosis of acute myocardial infarction in childhood. Am J Cardiol.

